# Autopsy of Adult Patients Deceased in an Academic Hospital: Considerations of Doctors and Next-of-Kin in the Consent Process

**DOI:** 10.1371/journal.pone.0163811

**Published:** 2016-10-13

**Authors:** Britt M. Blokker, Annick C. Weustink, M. G. Myriam Hunink, J. Wolter Oosterhuis

**Affiliations:** 1 Departments of Pathology and Radiology, Erasmus University Medical Centre, PO Box 2040, 3000 CA Rotterdam, the Netherlands; 2 Department of Radiology, Erasmus University Medical Centre, PO Box 2040, 3000 CA Rotterdam, the Netherlands; 3 Departments of Radiology and Clinical Epidemiology, Erasmus University Medical Centre, PO Box 2040, 3000 CA Rotterdam, the Netherlands and Centre for Health Decision Science, Harvard T.H. Chan School of Public Health, Harvard University, Boston, United States of America; 4 Department of Pathology, Erasmus University Medical Centre, PO Box 2040, 3000 CA Rotterdam, the Netherlands; University of Manchester, UNITED KINGDOM

## Abstract

**Introduction:**

Hospital autopsies, vanishing worldwide, need to be requested by clinicians and consented to by next-of-kin. The aim of this prospective observational study was to examine how often and why clinicians do not request an autopsy, and for what reasons next-of-kin allow, or refuse it.

**Methods:**

Clinicians at the Erasmus University Medical Centre were asked to complete a questionnaire when an adult patient had died. Questionnaires on 1000 consecutive naturally deceased adults were collected. If possible, missing data in the questionnaires were retrieved from the electronic patient record.

**Results:**

Data from 958 (96%) questionnaires was available for analysis. In 167/958 (17·4%) cases clinicians did not request an autopsy, and in 641/791 (81·0%) cases next-of-kin did not give consent. The most important reason for both clinicians (51·5%) and next-of-kin (51·0%) to not request or consent to an autopsy was an assumed known cause of death. Their second reason was that the deceased had gone through a long illness (9·6% and 29·5%). The third reason for next-of-kin was mutilation of the deceased’s body by the autopsy procedure (16·1%). Autopsy rates were highest among patients aged 30–39 years, Europeans, suddenly and/or unexpectedly deceased patients, and tissue and/or organ donors. The intensive care and emergency units achieved the highest autopsy rates, and surgical wards the lowest.

**Conclusion:**

The main reason for not requesting or allowing an autopsy is the assumption that the cause of death is known. This is a dangerous premise, because it is a self-fulfilling prophecy. Clinicians should be aware, and communicate with the next of kin, that autopsies not infrequently disclose unexpected findings, which might have changed patient management.

Mutilation of the deceased’s body seems a minor consideration of next-of-kin, though how it really affects autopsy rates, should be studied by offering minimally or non-invasive autopsy methods.

## Introduction

### Background

Autopsies on in-hospital deceased patients are performed to confirm, revise or identify the cause of death and relevant pathology, in order to provide clinicians with appropriate feedback on diagnosis and treatment. Despite the use of advanced diagnostic technologies in modern medicine, autopsies still reveal major diagnostic errors [1, 2]. Although the clinical autopsy thus remains an important health care quality control measure, the last 30–40 years have witnessed a worldwide decline of clinical autopsies [3–5]. Particularly in developed countries, with traditionally high autopsy rates, where financial and technical resources are available.

For a clinical autopsy, consent from next-of-kin is compulsory in most countries. The reluctance of next-of-kin to consent to autopsy, for example due to fear of mutilation of the body or concerns about organ retention of their loved ones [6–8], may be one explanation for low autopsy rates. Moreover, there seems to be a declining interest in autopsies among both clinicians and pathologists [9]. Although many clinicians still recognize the importance and benefits of autopsies [10, 11], in practice they find it difficult to request consent for autopsy, and often do not ask for it [10, 12]. In such circumstances, the next-of-kin will rarely consider the possibility of an autopsy [3].

### Purpose

The aim of this prospective observational study is to examine how often and why clinicians do not request an autopsy, and how often and for what reasons the next-of-kin allow or refuse it. We investigate the correlations between autopsy rate and certain patient characteristics and clinical aspects.

## Materials and Methods

### Study population and study design

For this prospective observational study, a survey was carried out at the Erasmus University Medical Centre, the tertiary referral centre, with around 1200 beds, for 3 to 4 million people in the Southwestern part of the Netherlands. Clinicians were asked to fill in a questionnaire about the consent process in their conversation with the next-of-kin subsequent to the death of an adult patient. According to the policy at Erasmus MC they should always offer the next-of-kin the possibility of an autopsy. For the purpose of our study, we had them ask the next-of-kin about their reasons for either giving or refusing consent to an autopsy in the ensuing conversation. It was deemed unethical to confront the bereaved with a separate questionnaire on this matter, which they had to fill out themselves immediately after the loss of a loved one. Thus the next-of-kin were not aware that their answers to questions of the doctor were collected to investigate their reasons for allowing or refusing an autopsy. This approach was considered acceptable because the questionnaire (S1 Fig), designed in consultation with the clinicians, was only meant for guidance and documentation of the conversation that clinicians at Erasmus MC normally have with next of kin upon demise of a patient.

As alluded to above, before starting the survey, we had some clinicians test the preliminary version of the questionnaire, which we adjusted according to their comments, before finalizing and implementing it. To boost clinicians’ compliance, we presented the study at their research meetings, explaining them the purpose of the study, and how to they should use the questionnaire to document their conversation with the next of kin. During the survey we reminded them of the study, by giving a second round of presentations. Also, as a standing reminder for the clinicians, we had the questionnaires added to the compulsory forms to be completed upon demise of a patient.

From January 2012 to April 2013, questionnaires were collected from 1000 consecutive cases.

Only patients who had died in-hospital from a supposed natural cause of death were eligible. Excluded were all deceased under age 18, those who underwent euthanasia, and victims of traffic accidents and crime. Case inclusion was based on the mortuary logbook, in which all in-hospital deceased patients are registered. As mentioned consent from next-of-kin for this study was not obtained, therefore, all patient information was anonymized and de-identified prior to analysis. Approval of the Erasmus MC Institutional Review Board and Ethics Committee was obtained for this purely observational study.

### Data collection

For each case we collected information on the consent process, the patients’ characteristics and clinical factors. The information provided in the questionnaires was checked, and if possible, missing data was retrieved from the electronic patient record (EPR). If nonetheless the information was insufficient, or unclear, the clinicians were contacted as soon as possible. In general they appeared very co-operative in providing the missing data.

A number of potentially relevant variables, partially based on the literature[4], was selected for registration in this study: patient characteristics (sex, age, ethnicity, religion, marital status) and clinical aspects, such as being a donor, the ward where the patient died, the way of dying (an unexpected or sudden death/ death after being ill for a longer period of time, a so called “long illness”), and who decided on consent for autopsy (partner/relatives/non-relatives).

Outcome measures were defined as: consent for autopsy requested (yes/no); consent for autopsy given (yes/no); the motivation for either decision; autopsy performed (yes/no). An autopsy had at least to include examination of thorax and abdomen.

Multiple-choice questions, based on the literature [6, 7, 10–12], were used to trace the considerations behind the decisions of clinicians and next-of-kin. Per multiple-choice question one or more motives could be given. A final, open question for “other motives” gave the option to enter any motives not yet addressed.

If a case record in the EPR mentioned “*autopsy not permitted*”, this was interpreted as autopsy consent having been requested by clinicians and not given by next-of-kin. In such cases the motives of next-of-kin remained unknown. If the case record said “*no autopsy*”, or if autopsy was not mentioned at all, it remained unknown whether or not consent was requested, and whether next-of-kin had been given the chance to consider autopsy.

### Data analysis and presentation

All information obtained from the questionnaires and the EPR was entered in an SPSS database. Missing variables within a case were scored as ‘unknown’, and included in the analyses.

Autopsy rates were calculated for all cases combined, and for specific subgroups. Per clinical ward the total number of deceased, the number of autopsies requested, and the number of given consents were presented graphically.

Cases were not eligible for further analyses if all outcome variables on decisions and considerations concerning autopsy consent were missing. Per subgroup in the consent process the percentage of given motives was plotted.

Patient characteristics and clinical aspect outcomes were cross-tabulated. To this end they were sorted into three groups: the numbers of autopsies not requested, the number of autopsies requested but not performed (including two restricted autopsies, and one case in which family abroad could not finalize the consent by signing the consent form), and the number of autopsies requested and performed. For each variable the distribution of cases within these three groups was analysed by Chi Square tests. If necessary subgroups were combined to meet the criteria for a valid Chi Square test: 80% of the cells in the table should have expected frequencies greater than five, and all cells should have expected frequencies greater than one.

## Results

### Overall

**Fig 1:**
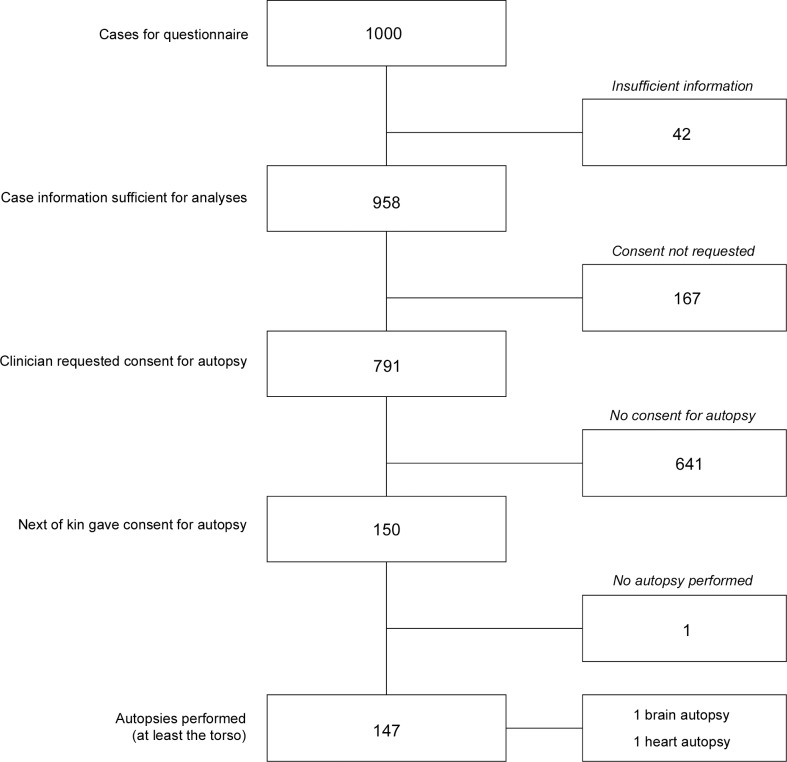
Flowchart survey. Based on the mortuary logbook 1000 consecutive cases of adult patients who had died in our academic hospital were included in this prospective observational study using a questionnaire. Information was deemed insufficient for further analyses if the clinician had neither reported in the questionnaire, nor in the electronic patient record, whether or not they had discussed autopsy with the next-of-kin and requested consent. Three consent procedures had to be discarded: two on restricted autopsies, and one because the next-of-kin were unable to sign the consent form.

In 958 of the 1000 cases the information gained from the collected questionnaires and the EPR was eligible for our analyses. In 873 of the 958 (91·1%) eligible cases the clinicians had filled in the questionnaire, in the 85 (8·9%) remaining cases the information on the consent process could be retrieved from the EPR. In 167 cases (17·4%) of these the clinician reported not to have requested consent for autopsy, and in 147 (18·6%) of the remaining 791 cases the next-of-kin consented to an autopsy including at least thorax and abdomen (see Fig 1), resulting in an overall autopsy rate of 14·7%.

### Autopsy percentages and distribution of cases

Among the 1000 cases, the highest overall autopsy rates measured per variable were 16·1% among women, 20·1% among deceased in the age group of 60–69 years old, 18·8% among deceased who had never been married, 16·7% among Europeans, 20·0% among Catholics, 15·2% among the sudden and/or unexpected deaths, and 40·0% among organ donors.

Considering only the 958 cases with information about the consent process, the measured autopsy rates were slightly different. Table 1 shows these cases and their distribution across the outcome measures concerning autopsy request and consent, per patient characteristic and clinical aspect.

**Table 1:** Available patient characteristics and clinical aspects.

		Autopsy rate	Autopsy torso performed	Autopsy torso not performed	Autopsy not requested	P-value X^2^-test
		N = 958	N = 147	N = 644	N = 167	(df)
***Sex***					** **	P = 0.650(2)
	male	14.5%	85	399	102	
	female	16.7%	62	245	65	
***Age group***					** **	P = 0.004*(12)
	18–29 years	8.3%	2	13	9	
	30–39 years	22.2%	6	15	6	
	40–49 years	20.6%	14	40	14	
	50–59 years	14.2%	24	116	29	
	60–69 years	20.9%	58	178	41	
	70–79 years	13.9%	32	162	36	
	80–99 years	6.7%	11	120	32	
***Marital status/ Partner***					** **	P = 0.328(10)
	Not/ Never married	19.4%	27	84	28	
	Partner	15.5%	9	37	12	
	Married	16.3%	92	385	87	
	Widow(er)	11.1%	6	39	9	
	Divorced	10.0%	3	21	6	
	Not registered/ Unknown	8.8%	10	78	25	
***Ethnicity***					** **	P = 0.005*(8)
	European	17.4%	137	524	127	
	Dutch Antilles, Aruba and Suriname	12.2%	6	35	8	
	Arabic	0%	0	25	7	
	Asian	7.7%	2	17	7	
	Other/ Unknown	3.2%	2	43	18	
***Religion***					** **	P = 0.080*(8)
	Christian	16.8%	28	116	23	
	Muslim	0%	0	28	11	
	Other	7.1%	1	10	3	
	None	18.3%	24	90	17	
	Unknown	15.5%	94	400	113	
***Way of dying***					** **	P = 0.033(2)
	Sudden/ Unexpected	15.8%	75	302	97	
	Long illness	14.9%	72	342	70	
***Donation***					** **	P <0.001*(2)
	No donation	14.3%	129	620	152	
	Any donation	31.6%	18	24	15	

* Expected frequencies did initially not meet criteria for valid Chi Square test, significance level was similar with combined subgroups.

The measured autopsy rates derived from this table are still highest among all the subgroups mentioned above, apart from the subgroup of ages. The autopsy rates in the subgroup of 30–39 years of age are the highest with 22·2%. The Chi Square tests showed that the outcomes of the consent process were unequally distributed over some of the variables.

**Fig 2:**
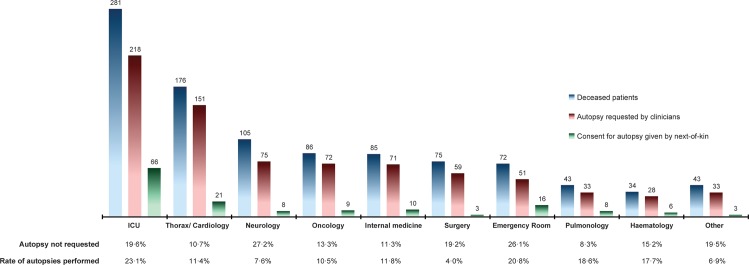
Autopsy requests and consents per hospital ward. For some of the deceased patients it is unknown whether the clinicians had requested consent for autopsy: from the Intensive Care Units (ICUs) 10, Thoracic surgery/Cardiology and its ICU 7, Neurology/Neurosurgery 2, Medical and Surgical Oncology 3, Internal Medicine 5, Surgery (general and all subspecialties) 2, the Emergency Room 3, Pulmonology 7, Haematology (including haemato-oncology) 1, and the other wards 2. For the overall rates of performed autopsies per ward we divided the number of autopsies actually performed by the total number of deceased patients. Autopsies had to include at least examination of thorax and abdomen. Two autopsies, respectively from the ICU and Cardiology were restricted to a single organ. One autopsy on a case of the emergency room was cancelled, because the next-of-kin were unable to sign the consent form.

The distribution of all 1000 cases per ward is shown in Fig 2. According to the Chi Square test, the outcome measures (autopsy not requested, autopsy not performed, autopsy performed) within the 958 cases were unequally distributed across the different wards (P<0·001, df = 18).

### Motives for decisions on autopsy consent

**Fig 3:**
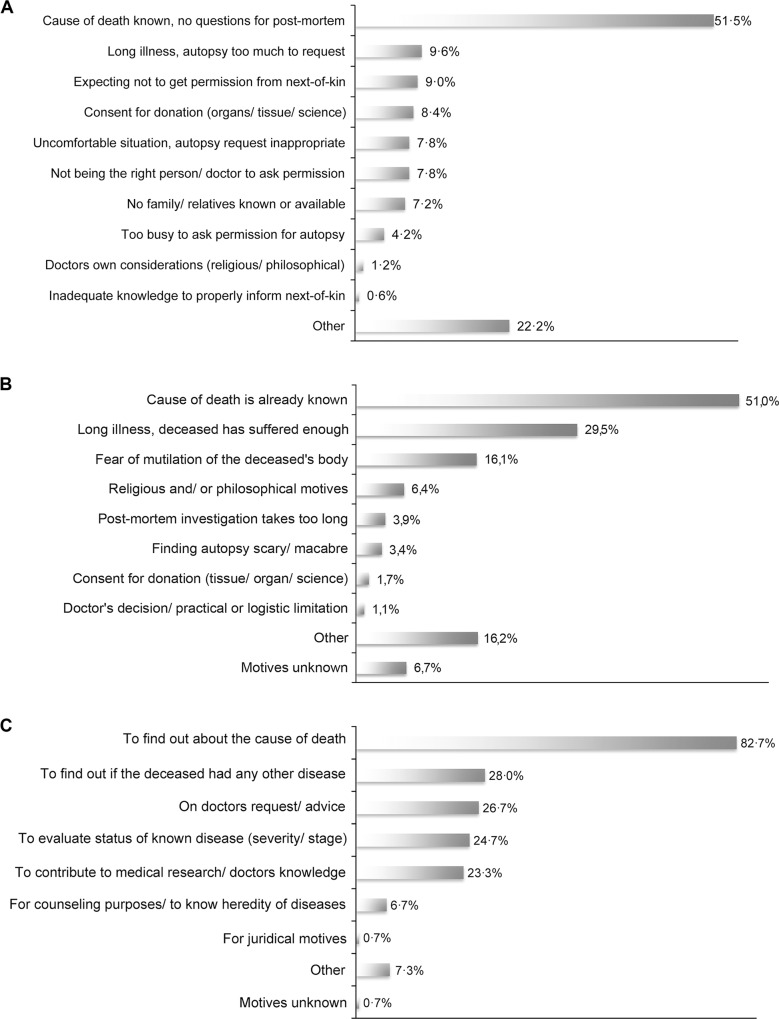
Considerations behind the decisions made in the consent process. Motives of clinicians and next-of-kin were investigated using a questionnaire with multiple-choice questions and a slot for free text. Per multiple-choice question one or more motives could be given. A. Clinician’s motives to not request consent for autopsy (N = 167). B. Next-of-kin’s motives to not give consent for autopsy (N = 641). C. Next-of-kin’s motives to give consent for autopsy (N = 150).

The main motive of clinicians to not request autopsy was a ‘*supposed known cause of death*’ (Fig 3A). This motive was mentioned in 86 of the 167 (51·5%) cases. Their second motive, ‘*a long illness after which an autopsy would be too much to request’* was mentioned in only 16 (9·6%) cases, in nine (5·4%) it was combined with the first motive. In 15 (9·0%) cases clinicians did not request an autopsy because of ‘*their expectation not to get consent from next-of-kin*’.

In 12 (7·2%) cases, which were not victims of traffic accidents, the conversation with next-of-kin was done by forensic doctors, who had been consulted for legal reasons. Being externally employed, they did not know about the autopsy policy in our institute and therefore did not ask permission for clinical autopsy. As motives for not requesting autopsy in these cases, we scored *‘other’* and *‘not the right person to ask permission’* for autopsy.

The two main motives of next-of-kin to not give consent for autopsy were similar to those of the clinicians (Fig 3B). A ‘*supposed known cause of death*’ was mentioned in 327 of the 641 (51·0%) cases, and ‘*a long illness whereby the deceased had suffered enough’* was mentioned in 189 (29·5%) cases. A combination of these two motives (with or without another motive) was reported in 70 cases (10·9%). Their third motive, ‘*fear of mutilation of the deceased’s body’*, was mentioned in 103 cases (16·1%). The combination of ‘*fear of mutilation*’ and a ‘s*upposed known cause of death*’ was reported in 42 (6·6%) cases, and the combination of ‘*a long illness*’ and ‘*fear of mutilation*’ in 32 (5·0%) cases.

‘*To find out about the cause of death*’ was the most important motive for next-of-kin to give consent for autopsy (Fig 3C). It was reported in 124 of the 150 cases (82·7%), followed by 42 (28·0%) cases in which next-of-kin wanted ‘*to find out if the deceased had any other disease’*. In 35 (23·3%) cases both of these motives were reported. In 40 (26·7%) cases the next-of-kin had decided to give consent for autopsy, because the clinician had requested it and/ or advised to have an autopsy performed.

## Discussion

### Main findings

Autopsy rates were the highest among patients who had died suddenly and/or unexpectedly, were on an intensive care unit, 30 to 39 years, European, or a donor. The main motive for clinicians to not request an autopsy, and for next-of-kin to not consent to it, was the assumption that ‘*the cause of death was known’*. Their second motive was ‘*a long illness’*, whereby clinicians found an autopsy too much to request and next-of-kin found that the patient had suffered enough. A third concern for next-of-kin was ‘*fear of mutilation of the deceased’s body’*.

### Limitations of this study

The study was conducted in a single academic institution where autopsies are encouraged to the extent that physicians should always offer the next-of-kin the possibility of an autopsy, and where there are no financial restraints for clinicians to request an autopsy. Therefore, the results may not directly apply to other hospitals with different autopsy policies. Nevertheless, the results are meaningful and in our view more generally applicable. Precisely because the study was carried out under conditions without financial and technical restraints it could trace considerations related to substance that may explain the present low autopsy rates. It is likely that where conditions and policies are less favourable towards autopsies, clinicians by similar considerations will feel even more justified to not pursue an autopsy.

In this survey the clinicians reported on the consent process in the final conversation they had with next-of-kin. The risk that this self-reporting method might introduce a bias towards desired answers was accepted, because it was for practical and ethical reasons, addressed under “Materials and Methods” in the paragraph “Study population and study design”, the only way to get the sought-after information. Assuming that the clinicians had always reported decisions in the consent process truthfully, we may conclude that they requested consent for autopsy in most cases (82·6%). In contrast, Burton and colleagues [13] found that consent for autopsy was requested in only 6·2% of eligible cases. In their study design, they did not investigate why clinicians did or did not request consent, because it might have introduced a bias. We believe that our results may indeed have been positively biased by our more extensive questioning and meticulous follow-up of the questionnaires, and also by the autopsy policy at our institute.

Among the patient characteristics, the Chi Square test did not show significant differences between religions, probably due to the high number of unknowns (63·4%). Probably the clinicians were reluctant to ask about religion, although the questionnaire included this item. Religion was more often reported in the EPR of patients who had suffered a long illness, than of those who had died suddenly and/or unexpectedly (respectively in 59% and 41%).

We were only able to evaluate univariable associations. Ideally, possible associations between variables and outcomes are evaluated with multivariable regression analyses, but to achieve a reasonable power for these analyses many more cases would have been necessary.

### Theoretical explanations and comparison with the literature

The overall autopsy rates on surgical and neurological wards were under 10%, and those of the ICUs and the emergency room above 20%. In Sheffield, UK, autopsy rates were reported to be below 10% for many specialties, including neurology and neurosurgery, but 11·6% for general surgery [13]. In Belfast, UK, the worst decline in autopsy rates was observed for surgical wards and ICUs, resulting in rates below 10%, whereas autopsy rates for neurosciences remained above 20% [12]. Apparently, attitudes and approaches of clinicians toward autopsy differ per specialty and hospital.

Several patient variables seem to influence the chance of an autopsy being requested and performed. Comparable to other studies [14, 15] autopsy rates were higher in younger patients, lowest in the age group of 80–99, and similar between the sexes. In contrast to another study [4] autopsy rates appeared not to be different depending on marital status or religion. Religious objections and concerns about mutilation have been described in several studies [6, 7, 10, 11]. Especially in Islam, removal of organs or disfigurement of the deceased’s body is generally forbidden [16]. In our cohort, not a single autopsy was performed on a deceased patient who was known to be a Muslim. In 48·7% of these cases next-of-kin had religious motives for refusing autopsy, and in 2·6% they feared of mutilation, compared to 4·8% and 13·8%, respectively, among known Christians.

Some of the considerations to not request or consent to autopsy should be addressed in order to improve autopsy rates. In this study ‘*inadequate knowledge about the autopsy procedure keeping clinicians from requesting consen*t’ [10] was mentioned in only a single case, complex consent forms were not mentioned as discouraging, neither were a decreased quality of the autopsy procedure or delay of the final autopsy report [8, 10, 12, 17].

In several cases both next-of-kin and clinicians mentioned that ‘*the deceased had suffered enough’* [6, 11, 18] which correlates to the lower autopsy rate we found among patients who died after a long illness. Perhaps fear of the discovery of misdiagnoses or treatment errors [6, 10, 12, 17] and the risk of malpractice suits [19] kept clinicians from requesting an autopsy in such cases. Or, more likely, both clinicians and next-of-kin had fewer unanswered questions than in cases of sudden death.

In general, clinicians tend to overestimate the reliability of advanced diagnostic technologies and therefore underestimate the value of autopsy [3, 4, 12, 20]. They assume that ‘*the cause of death is known’*, and may be unaware of the fact that there are still discrepancies found between premortem diagnoses and diagnoses found at autopsy [1, 2, 21–24]. If clinicians, when discussing the possibility of autopsy, tell the next-of-kin that the cause of death is already known and do not explain how or why an autopsy could still be of value, the next-of-kin will probably not consent to autopsy [25].

Improved knowledge and confidence will enable clinicians to ignore their ‘*expectation not to get consent from next-of-kin*’ and to always request consent for autopsy properly, or even motivate next-of-kin to have an autopsy performed. As a result, the next-of-kin are probably more willing to give consent [13, 14].

Improving the provided information about autopsies by clinicians and in the media may positively influence the attitude towards autopsy, and next-of-kin’s willingness to consent to autopsy. On a professional level, dedicated information forms could support clinicians’ requests for autopsy, especially if next-of-kin want to know what will be visible after an autopsy and whether they will be able to ritually prepare the deceased’s body for the funeral. From a different angle, changing the conventional, invasive autopsy technique may be the remedy for next-of-kin’s concerns about ‘*mutilation of the deceased’s body*’. Nowadays, non-invasive and minimally invasive autopsy methods are being developed for adults [26, 27] fetuses, and children [28]. The minimally invasive methods include post-mortem angiography and/or tissue biopsies, suitable for histology and/ or molecular diagnostics [29]. Higher autopsy rates may be achieved with these alternatives [30] although our study suggests a minor effect in view of the on average low percentage of next-of-kin refusing autopsy because of ‘*fear of mutilation of the deceased’s body*’. However in certain ethnical or religious subgroups non-invasive or minimally invasive procedures might significantly increase the acceptance of post-mortem investigation.

## Conclusions

Our study is the first to report that the main reason for not requesting or allowing an autopsy is the assumption that the cause of death is known. This is a dangerous premise because it is a self-fulfilling prophecy, and it ignores the value of the autopsy as a tool for quality control in medicine. Clinicians should be reminded that autopsies still disclose unexpected findings, which are significant for future patients.

Remarkably, mutilation of the body of the deceased seems a minor consideration of the next- of-kin, suggesting that minimally or non-invasive alternatives for the autopsy might not significantly alter autopsy rates. However, only if these alternatives are really offered will it be possible to study how they will affect autopsy rates in particular among populations with fundamental objections against the conventional autopsy, which thereby miss the benefits of post-mortem investigation.

## Supporting Information

S1 Fig Questionnaire translated into English. (PDF)

S1 Table Database in Excel. (XLSX)
