# Supplementary material for: Autopsy of Adult Patients Deceased in an Academic Hospital: Considerations of Doctors and Next-of-Kin in the Consent Process
Source: PLoS One. 2016 Oct 13;11(10):e0163811. doi: 10.1371/journal.pone.0163811 (PMC5063372; doi:10.1371/journal.pone.0163811)
Supplement: S1 Fig — (PDF) [file pone.0163811.s001.pdf]

Departments: Clinical Pathology and Radiology
MIA☎ +316 - 4844 2773

MIA- QUESTIONNAIRE

Form filled in by: ______________________________
Hospital ward: ________________ Doctor's position: ________________
Date: ____________

❶ **Patient specifics**

Sex: ☐ male ☐ female
Age: ______________________
Ethnicity: ______________________
Religion: ______________________
Donation: yes / no *
Way of dying: sudden / long illness *

Patient ID
(sticker)

* *Line through if **not** applicable*

❷ **Permission for autopsy requested?**

☐ yes → ❹ & ❺ *(backside)*

☐ no → ❸

❸ **Reason for not requesting permission for autopsy**

please tick as applicable

- ☐ Cause of death known, no questions for post-mortem
- ☐ Long illness, autopsy too much to request
- ☐ Expecting not to get permission from next of kin
- ☐ Consent for donation (organs/ tissue/ science)
- ☐ Uncomfortable situation, autopsy request inappropriate
- ☐ Not being the right person/ doctor to ask permission
- ☐ No family/ relatives known or available
- ☐ Too busy to ask permission for autopsy
- ☐ Doctor's own considerations (religious/ philosophical)
- ☐ Inadequate knowledge to properly inform next of kin
- ☐ Other: ______________________________

↓
end of form

Erasmus MC
Universitair Medisch Centrum Rotterdam

❹ **Relatives specifics**

Relation to deceased: ☐ partner ☐ family ☐ non-family
Ethnicity: ____________________
Religion: ____________________

❺ **Permission granted for conventional autopsy?**

☐ yes ↳ ❻

☐ no ↳ ❼

❻ **Motivation to permit autopsy** *please tick as applicable*

- ☐ To find out about the cause of death
- ☐ To find out if the deceased had any other disease
- ☐ On doctors request/ advice
- ☐ To evaluate status of known disease (severity/ stage)
- ☐ To contribute to medical research/ doctors knowledge
- ☐ For counseling purposes/ to know heredity of diseases
- ☐ For juridical motives
- ☐ Other: ____________________

❼ **Motivation to deny autopsy** *please tick as applicable*

- ☐ Cause of death is already known
- ☐ Long illness, deceased has suffered enough
- ☐ Fear for mutilation of the deceased's body
- ☐ Religious and/ or philosophical motives
- ☐ Autopsy takes too long
- ☐ Finding autopsy scare/ macabre
- ☐ Consent for donation (tissue/ organ/ science)
- ☐ Doctors decision/ practical or logistic limitation
- ☐ Other: ____________________

↓
end of form
